# Comprehensive dataset of global innovation index panel data (2013–2022): Clustering with K-means and principal component analysis

**DOI:** 10.1016/j.dib.2025.112194

**Published:** 2025-10-16

**Authors:** Edilvando Eufrazio, Helder Costa

**Affiliations:** a Instituto Nacional de Tecnologia, Av. Venezuela, 82, Rio de Janeiro, 20081-312, RJ, Brazil; b Universidade Federal Fluminense, Rua Passos da Pátria, 156, Bloco D, Escola de Engenharia, Niterói, 24210-240, RJ, Brazil

**Keywords:** Innovation, Clustering analysis, Dimensionality reduction, PCA, GII, Index, indicator

## Abstract

Over the last decade, innovation has become a focal point for policymakers, business leaders, and researchers worldwide. In that context, this dataset draws on the annual Global Innovation Index (GII), compiled by the World Intellectual Property Organization (WIPO) and available on the World Bank's Prosperity Data360 portal, to offer a refreshed view of national innovation landscapes. It covers 118 economies with complete data from 2013 through 2022, organized into seven core pillars: Institutions, Human Capital and Research, Infrastructure, Market Sophistication, Business Sophistication, Knowledge and Technology Outputs, and Creative Outputs. Although the dataset also includes overall GII scores and Innovation Input/Output Sub-Indices for each year, those aggregated measures were not used in clustering or Principal Component Analysis (PCA) to preserve the detail of the seven pillars.

To identify economies with similar innovation characteristics, we used the K-means algorithm, and the Elbow Method showed that five clusters worked best. We then applied this five-cluster framework in four different ways: focusing on input pillars only, focusing on output pillars only, combining all pillars, and using a version enhanced by Principal Component Analysis (PCA). PCA was introduced to reduce dimensionality and sharpen the divisions between clusters, which led to additional cluster labels for each scenario.

Because it offers both breadth and depth in its indicators, this dataset can be especially helpful for those examining how different nations innovate, gauging where they stand in comparison to peers, or investigating longer-term trends. This dataset is particularly helpful for researcher, policymaker, or professional seeking solid data on innovation; the information here can inform strategic thinking and support evidence-based decision-making.

Specifications Table

**Table:** 

Subject	Management of Technology and Innovation.
Specific subject area	Quantitative analysis of global innovation metrics using clustering and dimensionality reduction methods.
Type of data	Table, raw, Analysed.
Data collection	The dataset from the Prosperity Data360 portal of the World Bank includes 118 economies (2013–2022) across seven pillars. Data were normalized using StandardScaler (mean=0, std=1). We applied K-means clustering in three ways (inputs, outputs, and their combination) and repeated it with Principal Component Analysis (PCA) to reduce dimensionality. The Elbow Method determined five clusters, added as supplementary columns. Aggregated indices were not used in clustering and PCA, but they were included in a panel format to allow for time-based studies and to broaden research options.
Data source location	The data is hosted on the Prosperity Data360 portal of the World Bank (https://prosperitydata360.worldbank.org/en/dataset/WIPO+GII)
Data accessibility	Repository name: Mendeley Data Data identification number: 10.17632/xrr862ssjd.1 Direct URL to data: https://data.mendeley.com/datasets/xrr862ssjd/1
Related research article	none.

## 1 Value of the Data

• These data are useful in understanding global innovation patterns by clustering 118 economies across seven core innovation pillars, offering insights into national strengths and gaps in innovation performance.
• Researchers, policymakers, and business professionals can benefit from this dataset to benchmark innovation performance, assess policy impacts, and design strategies to innovation ecosystems.
• The dataset supports comprehensive analyses, including univariate, bivariate, and multivariate methods. The provision of normalized data facilitates direct comparisons, while the PCA-enhanced clustering can aid in interpreting complex multidimensional relationships by reducing noise and highlighting underlying patterns.
• Panel data from 2013 to 2022 allows for long-term studies, making it possible to analyze trends over time and compare different regions and innovation clusters.
• The K-means clustering method sorts of economies into useful categories based on their inputs, outputs, and innovation factors, using both PCA and non-PCA approaches. This method adds flexibility for a wide range of research applications. This approach offers considerable analytical flexibility, as researchers can choose the most relevant clustering variables based on their specific research questions.
• Clusters that rely only on input pillars (Cluster_inputs, Cluster_PCA_inputs) help to find economies that have similar abilities to innovate, resources, and supportive conditions.
• Clusters based solely on output pillars (Cluster_outputs, Cluster_PCA_outputs) group economies with comparable innovation performance and achievements.
• Clusters that combine all input and output pillars (Cluster_both, Cluster_PCA_both) give a complete picture, grouping economies according to their overall innovation system characteristics.
• Additionally, the versions improved by Principal Component Analysis (PCA) (Cluster_PCA_inputs, Cluster_PCA_outputs, Cluster_PCA_both) might provide stronger or clearer groupings by simplifying the data and reducing overlap between the key factors.
• Having both direct K-means and PCA-enhanced K-means cluster labels gives users the chance to look at the global innovation landscape from different angles and choose the method that fits their analysis needs best.
• The dataset's comprehensive nature, with no missing values for the included 118 economies over the ten-year period, and its organization into a single panel table, significantly simplifies data management and longitudinal analysis.

## 2 Background

The main objective in creating this dataset was to capture a comprehensive view of how innovation capacities vary across different economies over time. This resource covers 118 economies from 2013 through 2022, drawing on the annual Global Innovation Index (GII) produced by the World Intellectual Property Organization (WIPO) [1] and hosted on the World Bank’s Prosperity Data360 platform [2]. It is structured around seven pillars—Institutions, Human Capital and Research, Infrastructure, Market Sophistication, Business Sophistication, Knowledge and Technology Outputs, and Creative Outputs—thereby highlighting both the inputs and outputs of national innovation systems.

To make comparisons easier, each variable was adjusted to a common scale, and K-means clustering [3] was used to group economies with similar innovation characteristics. Principal Component Analysis (PCA) [4] was also used to simplify the data and uncover hidden patterns among the seven pillars. Principal Component Analysis (PCA)[4] was included to reduce dimensionality and reveal latent patterns among the seven pillars. In addition to the core indicators, the dataset retains overall GII scores and Innovation Input/Output Sub-Indices, although these were not employed in clustering or PCA.

This data article enhances research efforts by detailing the methods behind data collection and processing, offering a clear foundation for further studies. Researchers, policymakers, and professionals can readily adapt these data to investigate how innovation ecosystems evolve, benchmark international performance, or examine longitudinal patterns.

## 3 Data Description

This dataset is made available as a single Excel file named “gii_dataset_DIB.xlsx” [5], which is hosted on Mendeley Data. It contains a single worksheet, “Sheet1”, with 118 rows (excluding the header row), where each row corresponds to a specific economy, identified by its Economy Name and Economy ISO3 code. Annual indices from 2013 to 2022 are provided for the five input pillars of innovation—Institutions, Human Capital and Research, Infrastructure, Market Sophistication, and Business Sophistication—and for the two output pillars—Knowledge and Technology Outputs and Creative Outputs. Each column that contains a score is labeled by the corresponding year and pillar, for example, “2013_Institutions index,” “2014_Infrastructure index,” and so on. These indicators were originally sourced from the World Bank’s Prosperity Data360 portal, which compiles data from the World Intellectual Property Organization’s Global Innovation Index (GII).

**Fig. 1:**
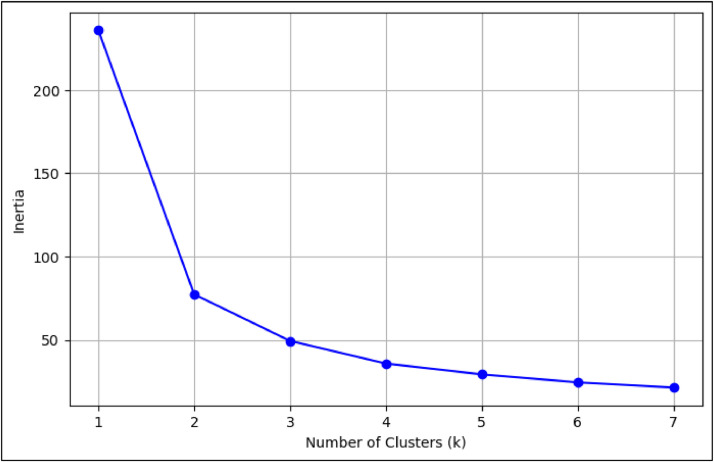
Elbow method graph considering outputs without PCA.

In addition to pillar-specific indices, the file includes Innovation Input Sub-Index, Innovation Output Sub-Index, and Global Innovation Index scores for each year, retained primarily for reference. To facilitate comparative studies, each entry has been normalized, and clustering was conducted using the K-means algorithm [6]. The Elbow Method helped determine that five clusters optimally capture the variation among economies (e.g. Fig. 1)[7]. For the PCA-enhanced clustering, Principal Component Analysis was applied to the same sets of pillar indicators (inputs, outputs, or all seven pillars, depending on the scenario) prior to K-means. This step aimed to reduce dimensionality and potentially enhance cluster separability by retaining approximately 80 % of the variance from the original variables, as detailed in the "Experimental Design, Materials and Methods" section.The clustering results were integrated as columns in the spreadsheet, naming them “Cluster_inputs”, “Cluster_outputs”, and “Cluster_both” for the direct K-means approach, and “Cluster_PCA_inputs”, “Cluster_PCA_outputs”, “Cluster_PCA_both” for the PCA-based method.

A summary of the key variables is provided in Table 1, highlighting each variable’s purpose, its description, and how it appears in the original dataset. This table helps readers locate specific columns of interest and understand how the data align with the conceptual pillars of the GII. In addition to the main pillar-specific indices, the file includes Innovation Input Sub-Index, Innovation Output Sub-Index, and Global Innovation Index scores for each year, retained primarily for reference.

**Table 1:** Overview of Variables, Descriptions, and Column Names in the dataset.

Variable	Description	Variable Structure Name
Economy Name	Name of the economy.	Economy Name
ISO Code	ISO code of the economy.	Economy ISO3
Institutions Index (2013–2022)	Annual index measuring the quality of institutions across years.	2013_Institutions index, …, 2022_Institutions index
Human Capital and Research Index (2013–2022)	Annual index for education and research infrastructure.	2013_Human capital and research index, …, 2022_Human capital and research index
Infrastructure Index (2013–2022)	Annual index for physical and technological infrastructure.	2013_Infrastructure index, …, 2022_Infrastructure index
Market Sophistication Index (2013–2022)	Annual index reflecting market dynamics.	2013_Market sophistication index, …, 2022_Market sophistication index
Business Sophistication Index (2013–2022)	Annual index of business environment and sophistication.	2013_Business sophistication index, …, 2022_Business sophistication index
Knowledge and Technology Outputs Index (2013–2022)	Annual index for knowledge creation and technological output.	2013_Knowledge and technology outputs index, …, 2022_Knowledge and technology outputs index
Creative Outputs Index (2013–2022)	Annual index measuring creative and cultural outputs.	2013_Creative outputs index, …, 2022_Creative outputs index
Cluster (Inputs)	Cluster labels based on input pillars using K-means.	Cluster_inputs
Cluster (Outputs)	Cluster labels based on output pillars using K-means.	Cluster_outputs
Cluster (Combined)	Cluster labels based on combined pillars using K-means.	Cluster_both
PCA Cluster (Inputs)	Cluster labels for input pillars after PCA.	Cluster_PCA_inputs
PCA Cluster (Outputs)	Cluster labels for output pillars after PCA.	Cluster_PCA_outputs
PCA Cluster (Combined)	Cluster labels for combined pillars after PCA.	Cluster_PCA_both

We normalized each entry and applied two primary clustering approaches to facilitate comparative studies:

1. Direct K-means on (a) input pillars, (b) output pillars, and (c) both sets combined.
2. K-means with Principal Component Analysis (PCA) as a dimensionality reduction step before clustering, enhancing cluster separability and reducing noise [7,8].

**Fig. 2:**
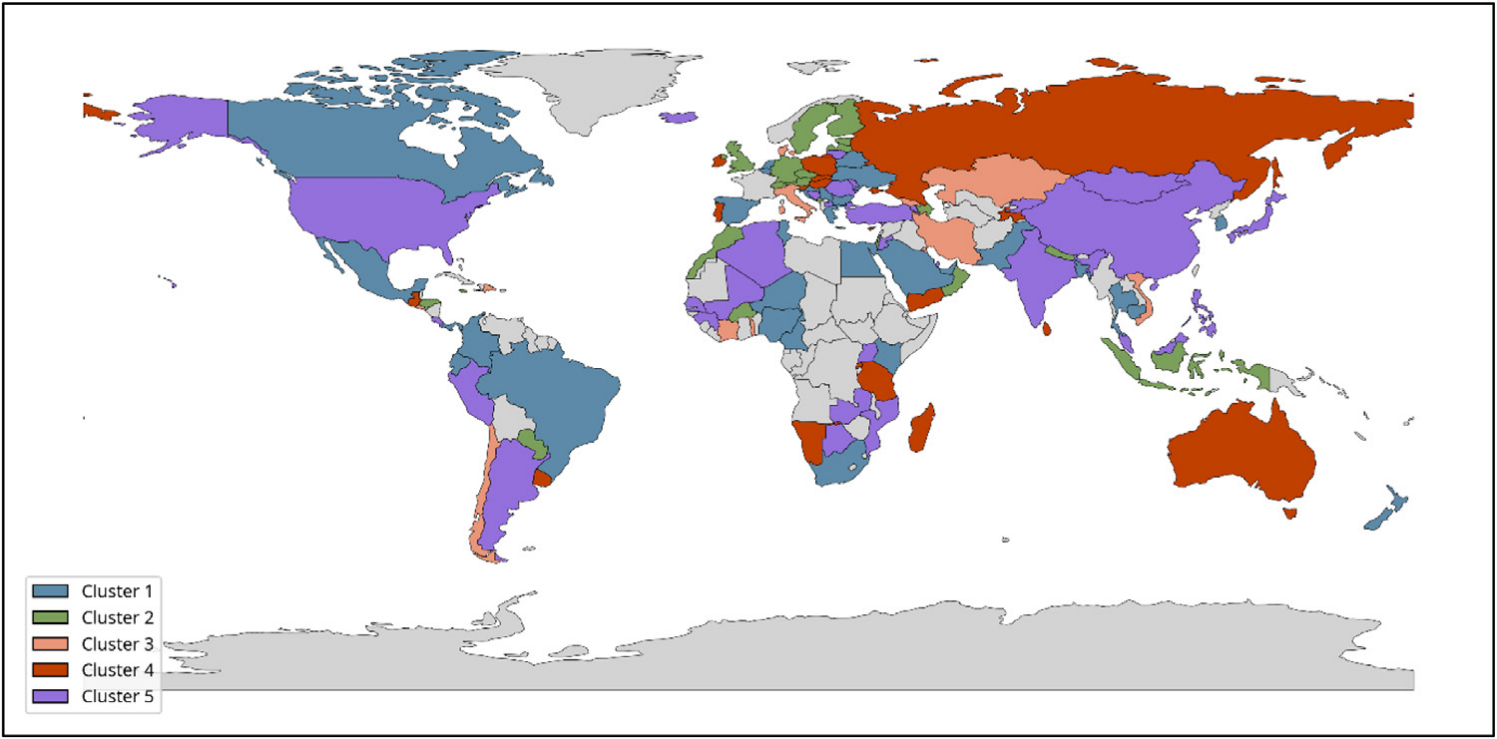
Country Clusters by GII Output Indicators.

Fig. 2 illustrates one example of cluster distribution for economies grouped by output indicators, providing a visual overview of how countries align under the Cluster_outputs configuration. The dataset, with its descriptive column names (e.g., 2013_Institutions index, 2020_Business sophistication index) and clear structure, supports both longitudinal and cross-sectional analyses.

Users can thus investigate how innovation profiles evolve over time and compare economies under different clustering strategies.

## 4 Experimental Design, Materials and Methods

To compile the dataset, we obtained annual Global Innovation Index (GII) indicators from the World Bank’s Prosperity Data360 portal, filtering and restructuring the raw data so that each row corresponded to a distinct economy and each column represented a combination of year and pillar (e.g., 2013_Institutions index). The steps included:

1. Data Acquisition
  • Downloaded the WIPO GII dataset from the Prosperity Data360 platform in Excel format.
  • Ensured that each subpillar (Institutions, Human Capital and Research, etc.) and each aggregated index (Innovation Input Sub-Index, Innovation Output Sub-Index, Global Innovation Index) were available for the selected years (2013–2022).
2. Data Preparation and Cleaning
  • Filtered rows to exclude any entry not identified as a Score.
  • Removed irrelevant columns and standardized the naming conventions of variables.
  • Compiled the data into a single table keyed by “Economy Name,” using a merging procedure to align indicators across years.
3. Clustering Procedure
  • Applied K-means clustering, first normalizing the selected pillars using StandardScaler from the scikit-learn library.
  • Optionally implemented Principal Component Analysis (PCA) to reduce dimensionality, retaining approximately 80 % of the variance before running K-means.
  • Used the Elbow Method to visualize and select an appropriate number of clusters, then appended the final cluster labels to the main dataset.
4. Implementation Details
  • Software and Libraries: Python 3.x, pandas[9], numpy [10], matplotlib [11], seaborn [12], scikit-learn[13].
  • Hardware: The analyses were performed on a standard personal computer (no specific hardware requirements are necessary due to the relatively modest dataset size).
  • Code Placement: The full Python script demonstrating data preparation, normalization, PCA integration, K-means clustering, and Elbow Method usage can be found below.

## Limitations

While this dataset spans a full decade (2013–2022) and includes 118 economies, it is important to note that its scope is defined by data availability. The original Global Innovation Index (GII) data, as referenced from sources like the World Bank, encompasses a broader list of economies (approximately 190, based on a comprehensive reference list considered for this study). For this dataset, however, we selected only economies with complete data available for all indicators across the entire 2013–2022 period. This selection process resulted in the exclusion of 72 economies that had missing indicators in one or more years within the specified timeframe. As a result, some countries for which GII metrics exist in certain years are not present in this consolidated panel dataset.

Minor inconsistencies can arise if economies update their data differently or apply varied methodologies over time, potentially affecting the annual comparability of scores. Although considerable effort was made to preserve data quality, any changes introduced by the original sources (e.g., WIPO or the World Bank) may influence the alignment of these indicators across years. Furthermore, the dataset focuses on quantitative measures, which may overlook qualitative aspects or localized nuances of innovation within individual economies. Researchers or practitioners aiming for more granular insights may need to consult additional sources or conduct primary data collection.

## Ethics Statement

The authors confirm that they have read and comply with the ethical guidelines for publication in Data in Brief. The dataset presented in this manuscript is derived solely from publicly available international indicators (World Intellectual Property Organization and the World Bank’s Prosperity Data360 portal). No human subjects, animal experiments, or social media data were involved in any stage of its collection or compilation. Consequently, informed consent, ethical committee approval, and animal welfare standards are not applicable to this work.

## Credit Author Statement

**Edilvando Eufrazio**: Conceptualization, methodology, data curation, writing original Draft, visualization, Project Administration. **Helder Costa:** Data Collection, Supervision, Writing – Review & Editing, Resources.

Both authors have reviewed and approved the final version of the manuscript.

## Data Availability

Mendeley DataGlobal_Innovation_Index_Clustered_Panel_ Data__With_PCA_2013_2022 (Reference data)
